# Does acute treatment of dapagliflozin reduce cardiac infarct size through direct cardiac effects or reductions in blood glucose levels?

**DOI:** 10.1186/s12933-020-01119-z

**Published:** 2020-09-19

**Authors:** Coert J. Zuurbier

**Affiliations:** Department of Anesthesiology, Amsterdam Cardiovascular Sciences, Laboratory of Experimental Intensive Care and Anesthesiology, Amsterdam UMC, University of Amsterdam, Amsterdam, The Netherlands

**Keywords:** SGLT2i, Hyperglycemia, Xylazine, Infarct size, Ischemia-reperfusion

In a recent article in this Journal, Lahnwong and co-workers [1], reported that acute pre-treatment of dapagliflozin just 15 min before start LAD occlusion reduced infarct size to a large extent in Wistar rats. These results are remarkable considering that most studies examining acute treatment with a SGLT2 inhibitor reported no effect on cardiac infarct size; only following chronic treatment (> > 1 d) do SGLT2i’s significantly reduce infarct size [2]. The question therefore arises what sets this study apart?

The answers may be found in the anesthetic regimen: animals were anesthetized with Zoletil and xylazine [1]. It is known that α_2_-adrenergic agonists such as xylazine or medetomidine acutely raise blood glucose to hyperglycemic values [3, 4] in non-fasted animals, through inhibition of insulin release from pancreatic β cells. Numerous studies have demonstrated that hyperglycemia is strongly associated with increased infarct size [5]. We therefore postulate that the intravenously administered dapagliflozin quickly lowered the high blood glucose levels in these animals, thereby explaining the reduction in infarct size by dapagliflozin in this experimental condition. It is therefore unfortunate that no information is provided by the authors on blood glucose, insulin, and ketone levels. We believe that in the search of potential cardioprotective mechanisms of SGLT2i’s, the nutritional status of the animal (fed versus fasted) and the substrate and hormone levels in the blood during the intervention being studied, should always be reported when examining these most promising antidiabetic drugs.

## Data Availability

Not applicable.
